# Embolizing pulmonary aspergillosis, mycobacterial & aspergillous splenic abscess and cytomegalovirus co-infection following steroid induced immunosuppression: a case report

**DOI:** 10.1186/s12879-018-3293-4

**Published:** 2018-08-06

**Authors:** Harsha Anuruddhika Dissanayake, Praveen Nilendra Weeratunga, Panduka Karunanayake, Rushika D. Lanerolle, M. V. Chandu de Silva, Saroj Jayasinghe

**Affiliations:** 1 University Medical Unit, National Hospital of Sri Lanka, Colombo, Sri Lanka; 2 Department of Clinical Medicine, Faculty of Medicine, University of Colombo, Colombo, Sri Lanka; 3 Department of Pathology, Faculty of Medicine, Colombo, Sri Lanka

**Keywords:** Aspergillosis, Fungal embolism, Acquired immune deficiency, Glucocorticoids, Splenic abscess

## Abstract

**Background:**

Aspergillosis is a serious infection particularly affecting the immunodeficient host. Its co-infection with tuberculosis and cytomegalovirus has not been reported before. Embolic events are well recognized with aspergillous endocarditis and aortitis. Splenic abscess is a rare serious complication of disseminated aspergillosis and is difficult to treat. We report the first case of multiple embolic events and splenic abscess in a patient with pulmonary aspergillosis and cytomegaloviral and tuberculous co-infection, without endocarditis or aortitis.

**Case presentation:**

Thirty-year-old male presented with fever and non-productive cough while on glucocorticoids for glomerulonephritis. He was found to have pulmonary aspergillosis and subsequently developed bilateral lower limb and cerebral fungal emboli and fungal abscess in the spleen. He had IgM and B cell deficiency and cytomegalovirus (CMV) and tuberculous co-infections. He recovered after prolonged course of antimicrobials, splenectomy and cessation of glucocorticoid therapy which also lead to the resolution of immune deficiencies.

**Conclusion:**

This report illustrates rare combination of B and T cell suppressive effects of glucocorticoids leading to co-infections with CMV, *Mycobacterium tuberculosis* and *Aspergillus* and systemic fungal embolization from pulmonary aspergillosis.

**Electronic supplementary material:**

The online version of this article (10.1186/s12879-018-3293-4) contains supplementary material, which is available to authorized users.

## Background

*Aspergillus* species cause serious infections in immunocompromised host. Common sites of infection are lungs and sinuses while central nervous system, cardiac valves and aorta are affected less frequently. *Aspergillus* species account for 28% of fungal endocarditis and are recognized to form large vegetations [1]. Embolizations to cerebral, lower limb, mesenteric and renal vasculature have been reported. *Aspergillus* aortitis is also known to cause distant embolic phenomena [2].

We report a patient with acquired immunodeficiency secondary to glucocorticoid use that developed simultaneous cytomegaloviral, tuberculous and pulmonary *Aspergillus* infections, complicated with multiple embolizations to cerebral and lower limb arteries and splenic abscess, without evidence of endocarditis or aortitis. Possible mechanisms of disease are discussed.

## Case presentation

A thirty-year-old male presented with fever and non-productive cough for 2 weeks with exertional dyspnoea. He had hypertension and renal impairment due to mesangioproliferative glomerulonephritis, diagnosed 3 months prior to current presentation, and was on bisoprolol, prazosin and prednisolone (0.5 mg/kg/day). Renal biopsy had not shown evidence of vasculitis.

On examination he was emaciated (BMI 18 kg/m^2^), febrile (38.2 °C), and had coarse crackles over right upper lung. Other system examinations were unremarkable.

The patient had a pancytopaenia with neutropaenia (neutrophil count 780 / mm^3^) and dysplastic hypoproliferative bone marrow. Peripheral blood detected cytomegalovirus (29,000 copies per microliter by PCR) and anti-CMV IgM was positive. After 21 days of ganciclovir (100 mg daily IV), CMV viral load became undetectable and pancytopaenia was corrected.

**Fig. 1:**
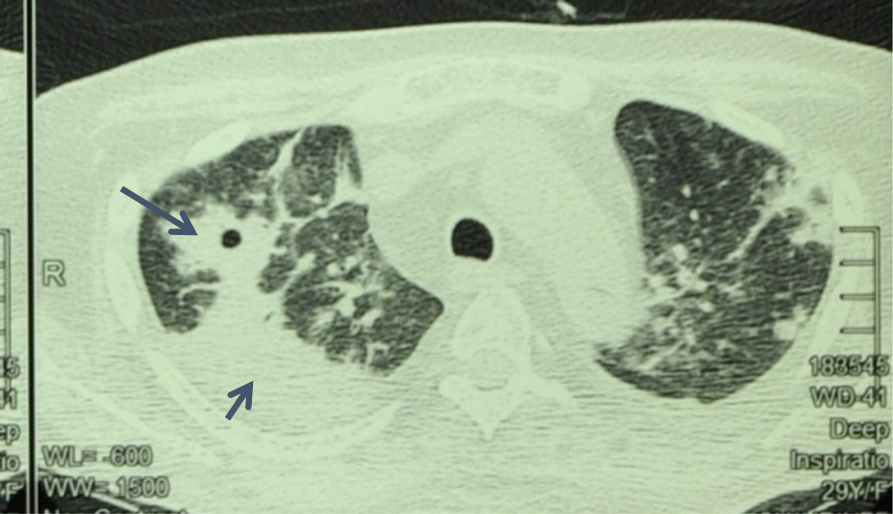
Contrast CT chest showing consolidation nodules and cavitation of upper lobe of the right lung (long arrow: cavity, short arrow - consolidation)

Contrast enhanced CT-chest showed right upper lobe consolidation with cavities (Fig. 1). Sputum smear for acid fast bacilli, culture and PCR for mycobacteria (Xpert MTb/RIF) were negative. However, tuberculosis PCR (IS6110 method) performed on his bone marrow aspirate was positive. Therefore, anti tuberculous therapy with isoniazid, rifampicin, ethambutol and pyrazinamide were commenced for disseminated tuberculosis. But, the response was poor.. *Aspergillus fumigatus* was detected on bronchoalveolar lavage wet smear and culture. Both lavage and serum were positive for galactomannan antigen. Therefore voriconazole 500 mg twice daily (oral) was commenced.

**Fig. 2:**
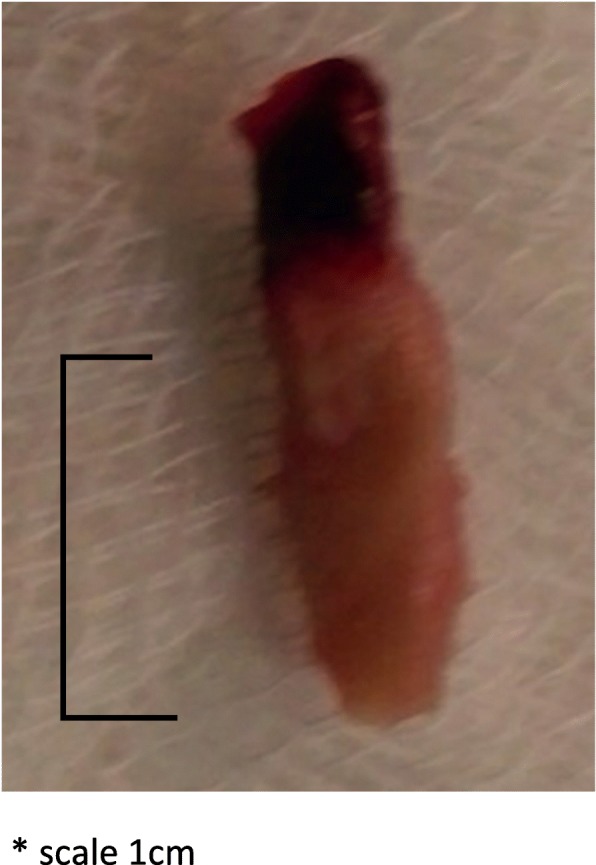
Embolus extracted from the right popliteal artery (scale 10 mm)

**Fig. 3:**
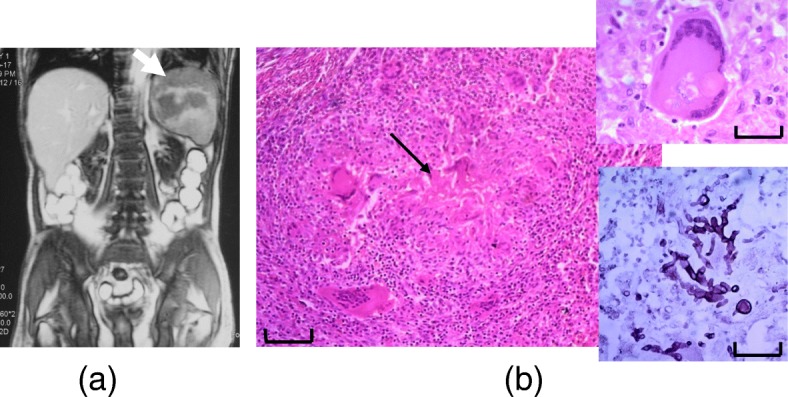
**a** Splenic abscess on MRI abdomen (white arrow). **b** Histology of splenic tissue demonstrating caseating granuloma (black arrow; in low power, scale 100 μm). Upper inlet shows granuloma with a multinucleated giant cell and lower inlet shows fungal filaments within granuloma demonstrated with Grocott stain in high power (scale 25 μm)

On sixth week of voriconazole, the patient developed acute bilateral lower limb ischaemia. Two emboli occluding bilateral popliteal arteries were extracted during emergency embolectomy (Fig. 2). Histology of emboli showed fungal filaments and culture isolated *Aspergillus fumigatus*. Trans-esophageal echocardiogram and magnetic resonance imaging (MRI) of the aorta were normal. However, MRI abdomen incidentally detected a large splenic abscess (Fig. 3a).

**Fig. 4:**
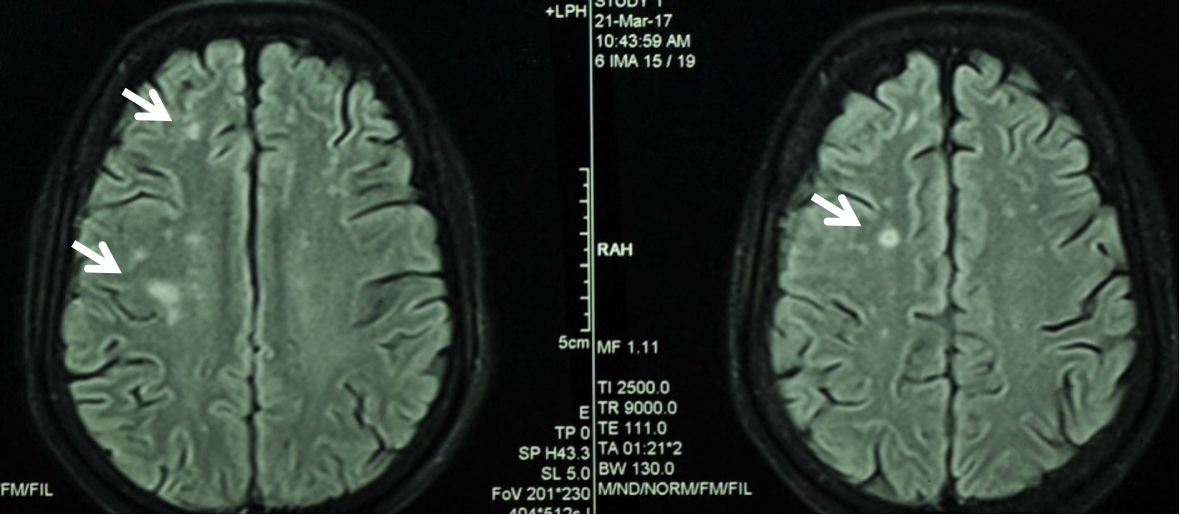
MRI brain showing multiple cortical infarcts (white arrows)

Despite treatment with voriconazole for further 2 weeks, the splenic abscess enlarged and treatment was switched to liposomal amphotericin 3 mg/kg/day intravenously. Four weeks later he developed acute left upper motor neuron type facial nerve palsy. MRI brain showed multiple small focal lesions in capsular and cortical regions suggestive of fungal embolism (Fig. 4). The neurological deficit completely resolved within a week.

Percutaneous aspiration under ultrasound guidance and 6 weeks of amphotericin failed to clear the splenic abscess. Therefore the patient underwent elective splenectomy. Histology of the splenic tissue showed fungal filaments within the abscess as well as caseating granulomas suggestive of tuberculosis of spleen (Fig. 3b).

Post-operative period was complicated with left Lower lobe pneumonia and parapneumonic effusion, from which he recovered with antibiotics and supportive measures. Subsequently, fever resolved and clinical status improved (see Additional file 1 for the timeline of disease evolution).

Due to the unusual constellation of infections (CMV, tuberculosis and aspergillosis) the patient was screened for an immune deficiency state which revealed B cell, IgM and IgA deficiencies and impaired T cell proliferation with concanavalin A (Table 1). Steroids were tapered and stopped 3 months after the current presentation. Six months later above immune defects returned to normal. Autoantibody profile (ANA, ANCA, rheumatoid factor and extracatable nuclear antibody profile) and HIV screening were negative on two occasions 4 months apart. Repeated imaging, bone marrow trephine and tumor screening with tumor markers did not identify any neoplasms.

**Table 1:** Summary of immune function assessment

	24 December 2016 (while on tapering steroids)	1 July2017 (6 months after steroid cessation)	Reference range
Total white cell count (per mm^3^)	5600	6700	4000–11,000
CD3 count (per mm^3^)	1866	1862	700–2100
CD4 count (per mm^3^)	580	760	300–1400
CD8 count (per mm^3^)	1306	1102	200–900
CD19 count (per mm^3^)	23	388	100–500
IgM level (mg/dL)	14	65	47–147
IgG level (mg/dL)	843	2283	569–1919
IgA level (mg/dL)	52	352	61–330
Nitroblue tetrazolium assay	Normal	–	–
T cell proliferation assay	Mild impairment	Normal	–

## Discussion

Disseminated invasive aspergillosis is a serious fungal infection particularly affecting the immune compromised individuals. Risk factors are immune deficiency either primary, or secondary to cytotoxic drugs, steroids, prolonged antibiotic use and haematological or solid organ malignancies. Structural lung disease and valve disease predispose to pulmonary aspergillosis and endocarditis respectively. This patient was on steroids, had myelodysplasia and severe B cell deficiency with IgM deficiency and T cell dysfunction with transient neutropaenia (that rapidly recovered with treatment of CMV). Immune response against *Aspergillus* is predominantly innate system dependent and neutrophil mediated [3]. Nevertheless, disseminated aspergillosis in a patient with selective IgM deficiency has been described before [4].

Multiple embolic events are known to occur in patients with *Aspergillus* endocarditis [1] and aortitis [2, 5, 6]. However, repeated trans-esophageal echocardiograms and magnetic resonance aortogram did not show valve defects, vegetations, aortitis or aneurysms. Therefore, we postulate that pulmonary aspergillosis was the primary focus from where fungal particles embolized through pulmonary veins and left heart into systemic circulation.

Splenic abscess was an incidental finding in our patient who did not have symptoms attributable to it. We think this was due to blood borne dissemination of *M. tuberculosis* bacilli, which was subsequently invaded by *Aspergillus* during a fungaemic phase. Caseating granuloma characteristic of tuberculosis was observed in splenic tissue (Fig. 3). Mycobacteria were not visualized microscopically or cultured from the abscess fluid, probably because 4 months of anti-tuberculosis therapy was completed by the time of splenectomy. Furthermore, *Aspergillus* has never been reported to cause caseating granulomas. Larger fungal embolization in to spleen with secondary tuberculous infection seems less likely due to the tortuous course of splenic artery and acute angle at the coeliac trunk origin. Furthermore, the patient never developed acute left hypochondrial pain suggestive of embolic splenic infarct. Fungal filaments were observed within the caseating granulomas in splenic tissue, which suggests co-infection of an existing caseating abscess. Although *Aspergillus* was never isolated from peripheral blood cultures, it does not rule out a fungaemia. Negative peripheral blood fungal cultures are not uncommon with disseminated fungal infection [7].

Disseminated tuberculosis would have formed splenic granulomas which were co-infected with *Aspergillus* during fungaemic phase. Complex alterations in immune system caused by glucocorticoids would have facilitated this unusual co-infection. Infections themselves can alter the immune response (e.g.: neutropaenia and T cell dysfunction due to CMV). This would also have contributed to the unusual co-infection.

Management of splenic abscess was challenging. Initial treatment with anti-fungals and percutaneous aspiration failed to clear the abscess necessitating splenectomy, despite the risks of surgical complications and further compromise of immunity. Voriconazole-rifampicin interaction and its poor penetration into abscess may have contributed to antifungal failure. Therapeutic drug monitoring (target trough level > 1 mg/L) is recommended when treating patients with voriconazole, especially when interacting drugs are co-prescribed [8]. Unfortunately, this facility was not available for us.

Assessment of the patient’s immune system revealed severe IgM deficiency, mild IgA deficiency, severe B cell deficiency and impaired T cell functions but normal T cell counts, neutrophil counts and functions on nitro-blue tetrazolium assay. Although glucocorticoids may give rise to some of the above abnormalities, IgM deficiency is not a recognized effect of glucocorticoid therapy [9, 10]. Nephrotic syndrome causes immunoglobulin deficiency. However this patient’s glomerular disease with subnephrotic proteinuria cannot explain B cell deficiency and predominant IgM rather than IgG deficiency.

Multiple severe unusual infections in this patient developed after commencement of glucocorticoids. Immune deficiencies completely reversed after its discontinuation. The patient had no features of immune defects prior to this illness. Therefore glucocorticoids are the most likely cause for the immune dysfunction. Impact of glucocorticoids on immune system are predominantly on T cells [11]. T cell dysfunction is a risk factor for CMV infection and was the likely culprit in our patient. Although steroids slightly reduce B cell numbers, immunoglobulin production is not affected in short term [9, 12]. B cells require assistance of T cells for normal immunoglobulin production. After years of glucocorticoid therapy, suppression of T cells can therefore reduce immunoglobulin synthesis. However, this occurs only with long term glucocorticoids therapy and IgA and IgG are the predominantly affected subtypes [13]. Therefore, observations in our patient are unusual, as the B cell depletion was marked, and it developed within few months of steroid therapy and, IgM was predominantly affected subtype. Glucocorticoids induced severe B cell depletion in our patient would have lead to IgM deficiency. No other factor could explain the transient immune dysfunction in the patient.

CMV infection itself is known to suppress T cell mediated immunity by TNF-alfa mediated expression of arachidonic acid and prostaglandin-E2 from infected monocytes [14]. This extreme state of T cell dysfunction with glucocorticoids and CMV infection would have predisposed him to develop disseminated tuberculosis. However, CMV infection activates plasmacytoid dendritic cells which increase proliferation and activation of B cells [15]. Therefore, CMV infection does not explain our patient’s severe B cell deficiency.

## Conclusion

This case highlights several features of interest. First, to the best of our knowledge, this is the first case of concomitant CMV, tuberculous and aspergillous infection in a transient state of immunosuppression. Second, our patient developed multiple fungal emboli from a pulmonary focus of aspergillosis, a phenomenon not described before. Third, the patient had a severe B cell and IgM deficiency, which reversed after discontinuation of glucocorticoids. This effect has not been described before and therefore we highlight the need for further studies on effects of glucocorticoids on human immune system. Finally, this case illustrates the importance of judicious use of glucocorticoids being aware of their wide range of effect on human immune system.

## Additional file

Additional file 1: Timeline. Graphical representation of temporal evolution of the disease. (DOCX 38 kb)

## Abbreviations

ANA: Antinuclear antibodies
ANCA: Anti neutrophil cytoplasmic antibodies
BMI: Body mass index
CMV: Cytomegalovirus
CT: Computed tomography
dsDNA: double stranded deoxyribonucleic acid (antibodies)
HIV: Human immunodeficiency virus
MRI: Magnetic resonance imaging
PCR: Polymerase chain reaction
