# Supplementary material for: Embolizing pulmonary aspergillosis, mycobacterial & aspergillous splenic abscess and cytomegalovirus co-infection following steroid induced immunosuppression: a case report
Source: BMC Infect Dis. 2018 Aug 6;18:367. doi: 10.1186/s12879-018-3293-4 (PMC6080400; doi:10.1186/s12879-018-3293-4)
Supplement: Supplementary file 1 — Timeline. Graphical representation of temporal evolution of the disease. (DOCX 38 kb) [file 12879_2018_3293_MOESM1_ESM.docx]

30 year old male

Hypertension & stage III CKD (diagnosed 2016 June)

Mesangioproliferative glomerulonephritis

On bisoprolol, prazosin, prednisolone

Pancytopenia, cavitating pneumonia in right lung upper zone, peripheral blood CMV PCR 29000/mm^3^, bone marrow dysplasia and positive TB PCR. Sputum AFB and TB culture: negative.

Pale, febrile, dyspnoeic, right upper lung coarse crackles

Fever, dry cough, exertional dyspnea x 2 weeks

**20.10.2016**

Ganciclovir IV, anti TB therapy.

Prednisolone tailed off.

Pancytopenia corrected, viremia cleared.

**04.03.2017**

Acute left UMN facial palsy. MRI : cerebral emboli

Enlarging splenic abscess, ongoing fever

Liposomal amphotericin B IV

**05.02.2017**

**20.01.2017**

Bilateral acute lower limb ischaemia.

Popliteal emboli cultured *A. fumigatus*. Normal cardiac valves and aorta. Splenic abscess on MRI.

Severe B cell, IgM deficiency and T cell dysfunction.

**01.12.2016**

Persistent fever, worsening respiratory symptoms, poor radiological resolution

**30.10.2016**

Diagnoses : CMV viremia, smear negative pulmonary tuberculosis with bone marrow dissemination.

Bilateral popliteal embolectomy

Voriconazle oral

Bronchial lavarge : *Aspergillus fumigatus*

**01.07.2017**

B cell count, IgM levels returned to normal

Fever resolved and clinically improved.

Splenectomy. Histology : caseating granuloma and *Aspergillus* filaments.

Poor resolution of splenic abscess, ongoing fever

**20.03.2017**

Follow up: afebrile, normal inflammatory markers, stable renal functions (CKD IV)

**Conclusion:**

**Glucocorticoid induced severe B cell and IgM deficiency, CMV sepsis, disseminated tuberculosis and aspergillosis causing lower limb and cerebral embolization**
